# Eco-Friendly Illite as a Sustainable Solid Lubricant in Calcium Grease: Evaluating Its Thermal Stability, Tribological Performance, and Energy Efficiency

**DOI:** 10.3390/ma19030464

**Published:** 2026-01-23

**Authors:** Maria Steffy, Shubrajit Bhaumik, Nabajit Dev Choudhury, Viorel Paleu, Vitalie Florea

**Affiliations:** 1 Department of Science and Humanities, Amrita School of Engineering, Amrita Vishwa Vidyapeetham, Chennai 601103, TN, India; mariasteffy39@gmail.com; 2 Tribology and Interactive Surfaces Research Laboratory (TRISUL), Department of Mechanical Engineering, Amrita School of Engineering, Amrita Vishwa Vidyapeetham, Chennai 601103, TN, India; 3 Department of Energy Engineering, Assam Science and Technology University, Guwahati 781013, AS, India; nabajit.astu.2017@gmail.com; 4 Mechanical Engineering, Mechatronics and Robotics Department, “Gheorghe Asachi” Technical University of Iași, 43 Prof. Dimitrie Mangeron Blvd, 700050 Iasi, Romania; 5 Department of Structural Mechanics, “Gheorghe Asachi” Technical University of Iași, 1 Prof. Dimitrie Mangeron Blvd, 700050 Iasi, Romania; vitalie.florea@academic.tuiasi.ro

**Keywords:** illite, castor oil, calcium hydroxide grease, tribological properties, thermal stability, energy consumption, sustainable greases, natural clay mineral

## Abstract

**Highlights:**

**What are the main findings?**

- Optimal illite concentration (0.1 wt.%) reduces friction and wear by 53% and 57%, respectively, when compared to calcium grease without the additive.
- Calcium grease with 0.1% illite exhibited a 93% higher apparent frictional energy density, a 47% lower wear intensity, a 21% higher load–wear index, and 6% lesser current consumption than CG.
- Illite increases the thermal stability of calcium grease (CG) up to 400 °C in a nitrogen atmosphere, but promotes oxidative degradation in an oxygen atmosphere.
- Illite’s layered structure provides helpful anti-wear and friction-reducing characteristics.

**What are the implications of the main findings?**

- Calcium greases containing illite particles can be a suitable alternative as an environmentally friendly grease.
- The findings support the goals of sustainable tribology and green engineering and promote the development of high-performance, eco-friendly green lubricants.

**Abstract:**

This study investigates the influence of the additive illite on the thermal, tribological, and energy efficiency characteristics of calcium grease (CG) at different concentrations (0.05 wt.%, 0.1 wt.%, 0.2 wt.%, 0.4 wt.%, 0.6 wt.%, and 0.8 wt.%). Thermo-gravimetric analysis under inert and oxidative atmospheres revealed that illite enhances thermal stability by increasing inorganic residue under N_2_, but promotes oxidative degradation under O_2_, limiting practical thermal use to around 400 °C. Grease with 0.1 wt.% illite (CGI2) performed well in tribological tests by reducing the coefficient of friction and wear scar diameter by 53% and 57%, respectively, compared to the base grease. Fleischer’s energy-based wear model showed that all grease samples operated within the mixed friction regime, and CGI2 exhibited a 93% higher apparent frictional energy density and a substantially lower wear intensity that was 47% lower than the base grease, indicating improved energy dissipation and wear resistance. All samples had the same weld load (1568 N), but CGI2 had a 21% higher load–wear index than the base grease in the extreme-pressure test, indicating better load-carrying capacity. In the energy consumption test, a 6% reduction in current consumption was observed in CGI2 in comparison with the base grease. Overall, illite at an optimal concentration significantly enhances lubrication performance, wear protection, and energy efficiency.

## 1. Introduction

In recent years, increasing environmental awareness and tightening regulations have intensified the search for sustainable alternatives to conventional lubricants. Traditional greases, typically derived from mineral oil and thickened with metallic soaps, often contain additives that pose risks to human health and the environment due to their toxicity and poor biodegradability [1]. As the demand for environmentally friendly lubricants continues to grow, the development of bio-based greases using renewable base oils, recycled base oils [2], and eco-friendly additives has become a significant area of research in tribology. Among bio-based options, castor oil has gained attention for its unique physicochemical properties, including high viscosity, excellent polarity, and a naturally occurring hydroxyl group that contributes to oxidative stability and superior film-forming capability [3,4,5,6,7]. Its triglyceride structure with ricinoleic acid allows for strong interaction with metal surfaces, making it a promising base oil in grease formulations. To improve its consistency and rheological behavior, a thickener is often employed. A calcium-based soap matrix enhances the grease’s high-temperature stability and water resistance [8]. However, even with such favorable properties, bio-based greases often require performance-enhancing additives to match or exceed the load-bearing, anti-wear, and friction-reducing characteristics of traditional greases. Naturally occurring minerals are good alternatives to conventional additives due to their non-toxic nature and cost-effectiveness [9,10]. According to reports, the tribological properties of base oil have been markedly enhanced by the addition of kaolin, a phyllosilicate, as a lubricating additive [11]. According to a number of reports, adding serpentine powder may have increased the wear resistance as well [12,13,14,15,16]. The anti-wear and anti-friction qualities of muscovite, another phyllosilicate, have also been used to enhance base oil [17,18,19,20]. When applied as an additive, sepiolite created a tribolayer on the surface that had been worn [21,22]. By creating a ttribolayeron the interacting surfaces, phyllosilicate appears to be a viable lubricant additive to lessen wear [23]. Additionally, the tribological qualities were improved by naturally occurring silicates with a slippery nature, such as mica and talc [24]. Furthermore, silicates were discovered to reduce pollutants and wear down particles, halting additional damage [25]. Clay minerals, especially layered silicates, have shown promise as environmentally friendly solid additives in grease systems due to their high surface area, lamellar structure, and chemical stability [26]. It was reported that the addition of Attapulgite clay to 150SN mineral oil significantly reduced the coefficient of friction and wear [27]. Studies shows that the use of Attapulgite and bentonite as grease thickeners enhances the tribological properties of the grease [28]. The addition of Antigorite to oil reduces the friction coefficient, wear volume, and power consumption of the driving motor [29]. It is shown that C20A Nano clay dispersions with montmorillonite act like gels with adjustable viscosity and viscoelasticity, showing excellent lubrication with friction and wear performance equal to or better than bentonite grease [6]. Illite, a naturally occurring non-expanding clay mineral, belongs to the mica group and has a structure similar to that of muscovite, composed of stacked tetrahedral–octahedral–tetrahedral (TOT) layers [30,31,32]. The weak interlayer bonding in illite, predominantly through electrostatic interactions and Van der Waals forces, allows for easy shearing between layers, making it a potential solid lubricant [33,34]. Additionally, illite possesses good thermal stability, low reactivity, and high abundance, rendering it a cost-effective and sustainable alternative to traditional solid additives such as molybdenum disulfide. The tribological behavior of illite is less explored compared to other lamellar materials. Its layered morphology and micro-rolling or exfoliating behavior under mechanical stress could provide its ability to reduce wear and friction [35,36]. Despite these promising attributes, the integration of illite into bio-based grease systems such as castor oil–calcium hydroxide grease has not been extensively studied. The synergy between illite and bio-based greases may offer a pathway to high-performance green lubricants.

This study aims to investigate the effect of illite as a solid additive on the tribological performance of calcium hydroxide-thickened castor oil grease to assess the potential of illite as an eco-friendly and cost-effective additive in bio-based grease formulations and to explore the structure–property–performance relationships governing its behavior under tribological stress. Experimental evaluation involves a comparative analysis between greases formulated with varying concentrations of illite and a control sample without additives. By combining a bio-based base oil with a naturally occurring mineral additive, this research aligns with the broader goals of sustainable tribology and green engineering.

## 2. Materials and Methods

### 2.1. Materials

**Figure 1:**
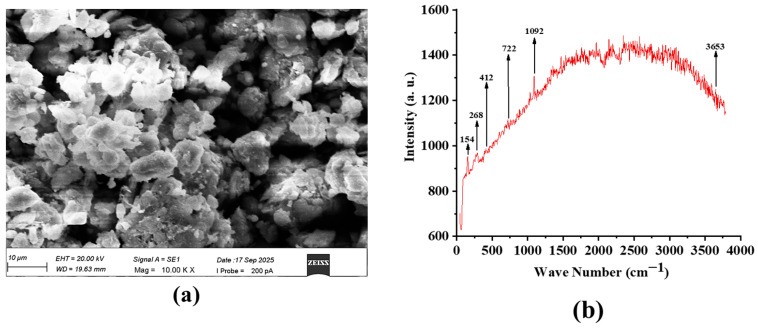
(**a**) SEM image and (**b**) Raman spectrum of the additive.

Calcium hydroxide, hydrogenated castor oil, and castor oil were acquired from Research-Lab Fine Chem Industries (Mumbai, India), Shree Chemicals (Ankleshwar, India), and a nearby oil mill in Chennai, India. Illite was purchased from Marudhar Impex, Ahmedabad, Gujarat, India. To study the morphology of illite, scanning electron microscopy (SEM) images were taken, as shown in Figure 1a. The average particle size of illite was measured as 3.12 ± 0.72 microns. The physicochemical properties of the base oil are given in Table 1. The balls used were made of EN31 steel, as confirmed by chemical composition analysis (Table 2). The hardness of the EN31 balls is 55 ± 1 HRC at 1 kg load. The roughness of the balls is 1.01 ± 0.1 µm.

The Raman spectrum of the sample shows peaks at 154 cm^−1^, 268 cm^−1^, 412 cm^−1^, 722 cm^−1^, 1092 cm^−1^, and 3653 cm^−1^ (Figure 1b). The low-wavenumber modes below 500 cm^−1^ correspond to lattice vibrations of the layered silicate framework [37], while the band at 722 cm^−1^ is attributed to Si–O bending vibrations [37]. A strong band at 1092 cm^−1^ arises from Si–O stretching within the tetrahedral sheets [37], which is a diagnostic feature of phyllosilicates. The sharp high-wavenumber band at 3653 cm^−1^ corresponds to structural Al–OH stretching vibration [37], confirming the presence of hydroxyl groups in the octahedral sheet. Together, these features are consistent with the Raman signature of illite, a 2:1 alumino-silicate mineral of the mica group [37].

### 2.2. Preparation of Calcium Grease

**Figure 2:**
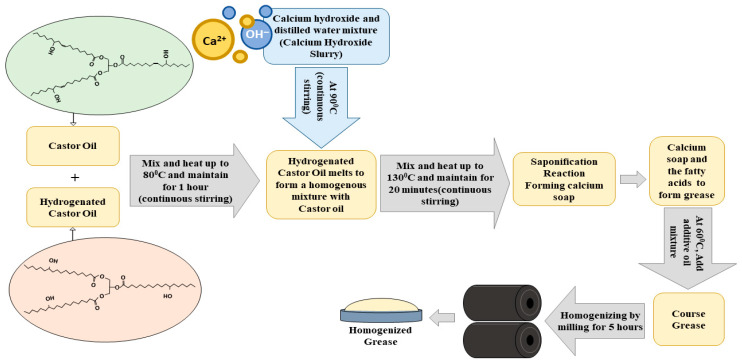
Preparation method of the calcium grease.

**Figure 3:**
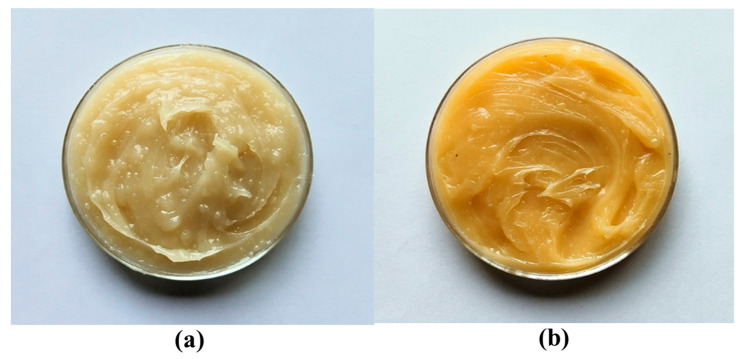
Pictographic image of (**a**) base calcium grease and (**b**) calcium grease with illite.

The method used by Negi et al. [12] was adopted for grease preparation in this work. The flow of the preparation is illustrated in Figure 2. By mechanically stirring 60 g of castor oil and illite, an additive base oil mixture was prepared. At 80 °C, 27 g of hydrogenated castor oil and 80 g of castor oil were stirred in a magnetic stirrer for one hour. Then, to initiate saponification, a mixture of distilled water (30 g) and calcium hydroxide (7 g) was added to it. Then, the temperature of the mixture was gradually increased to 130 °C and it was maintained at that temperature for 20 min. It was cooled to 60 °C and then the additive base oil mixture was added. The mixture was then cooled to room temperature. The obtained course grease was homogenized by rolling and milling with 39% total base oil for 5 h to obtain a well-structured refined grease. Figure 3 shows the prepared base grease and the illite grease. The following samples with different concentrations of the additive were produced: base calcium grease (CG), CG + 0.05 wt.% of illite (CGI1), CG + 0.1 wt.% of illite (CGI2), CG + 0.2 wt.% of illite (CGI3), CG + 0.4 wt.% of illite (CGI4), CG + 0.6 wt.% of illite (CGI5), and CG + 0.8 wt.% of illite (CGI6).

**Figure 4:**
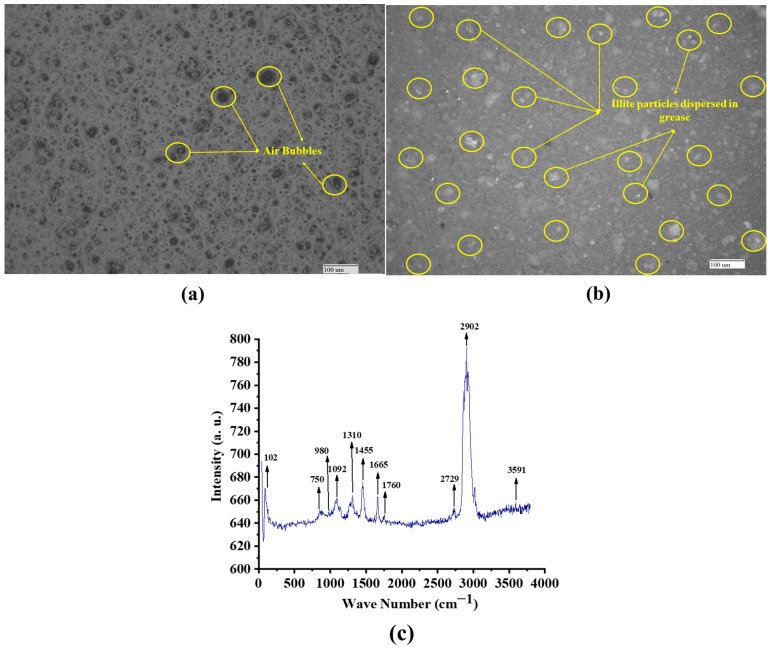
Optical microscope images of (**a**) CG, (**b**) CG with illite, and (**c**) Raman spectrograph of the CG containing illite.

The worked cone penetration of the base grease was measured at 257 dmm using ASTM D217 [38,39] with 60 double strokes, suggesting it is classified as NLGI grade 2. Figure 4 displays the Raman spectrograph of the illite-incorporated grease. The Raman peaks of the base calcium grease without illite have already been reported in the authors’ previous work [39]. As indicated, the base calcium grease is characterized by prominent fatty acid bands (2850–3000 cm^−1^ and 1430–1600 cm^−1^), ester bands (1000–1200 cm^−1^), and carboxyl groups (1600–1700 cm^−1^) [39]. In the present work, the grease has the Raman peaks for the carboxyl groups in the fatty acids (1600–1700 cm^−1^) [40], the ester linkages in the castor oil’s triglycerides (1000–1200 cm^−1^) [40] and the fatty acid components (2850–3000 cm^−1^ and 1430–1600 cm^−1^) [40]. The presence of illite was confirmed by the presence of its characteristic peaks at 750 cm^−1^ from Si–O bending vibrations, at 1092 cm^−1^ from Si–O stretching within the tetrahedral sheets, and at 3591 cm^−1^ from structural Al–OH stretching vibrations [40].

The drop point of the grease samples (Table 3) was evaluated as per the ASTM D566 standard [41]. A reduction in drop point was observed with the addition of illite. The reduction in drop point is due to the adsorption of calcium ions by the illite particles present in the grease [42].

### 2.3. Thermal Stability of the Samples

Thermal stability of the prepared grease samples was analyzed using differential scanning calorimetry analysis (DSC) (Make: Mettler Toledo, Greifensee, Switzerland, Model: DSC3) and thermo-gravimetric analysis (TGA) (Make: Mettler Toledo, Switzerland, Model: TGA2). The tests were conducted in both oxygen and nitrogen environments to understand the behavior of the samples. The grease samples were heated from 25 °C to 800 °C at the rate of 1 °C per 6 s.

### 2.4. Tribological Tests

**Figure 5:**
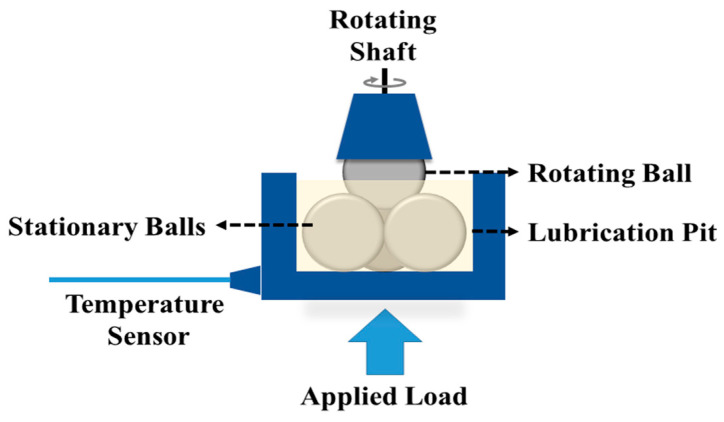
Schematic diagram of the four-ball tribometer.

The tribological qualities of the greases were determined by their coefficient of friction (COF), anti-wear properties, and extreme-pressure properties. A four-ball tribometer (Make: Magnum Engineers, Noida, India, Model: TE-800-FBT) was used to analyze these properties [43]. Although rolling or sliding bearing tests provide application-level performance validation, standardized four-ball tribological tests are widely employed as preliminary screening tools for evaluating the friction-reducing, anti-wear, and extreme-pressure characteristics of lubricating greases (ASTM D2266 [44], ASTM D5183 [45], and ASTM D2596) [46]. The four-ball configuration offers controlled point-contact conditions with high repeatability, enabling the systematic comparison of lubricant formulations and additive concentration effects. In the present study, four-ball testing was therefore selected to establish comparative tribological trends and to elucidate the lubrication mechanisms associated with illite addition. Figure 5 shows a schematic diagram of the four-ball tribometer. The anti-wear properties of the grease samples were analyzed as per the standard of ASTM D2266, wherein the fourth ball rotated at a speed of 1200 rpm under a 40 kg load at 75 °C for 1 h. The coefficient of friction was found according to the standard of ASTM D5183, wherein the fourth ball rotated at a speed of 600 rpm under a 40 kg load at 75 °C for 1 h. All of the experiments were repeated three times and the values were averaged. Optimized samples were selected based on the results of the above tests and these were then tested on their extreme-pressure properties according to the ASTM D2596 standard. In this analysis, each test lasts for 10 s and the fourth ball rotates at a speed of 1670 rpm under a fixed load. The load was increased in subsequent tests according to the load chart of the ASTM D2596 standard until the four balls welded together or the sight of smoke from the ball pit. Test parameters were given in Table 4 [39].

It is emphasized that four-ball tribological tests, although standardized and widely used for comparative screening, do not directly predict grease performance in rolling-element bearings, where contact kinematics, lubrication starvation and replenishment, and fatigue-driven failure mechanisms differ substantially from the point-contact sliding conditions of the four-ball configuration.

### 2.5. Analysis of Friction and Wear States Using Fleischer’s Wear Model

The Fleischer-model-based analysis complements conventional tribological metrics by providing an energy-oriented comparison, while its broader validation under component-level contacts has been addressed in the authors’ earlier study [47]. Fleischer’s energy-based wear model explains wear mechanisms and predicts wear in various systems by relating the wear to the friction that caused it [47]. It is based on the energy accumulation hypothesis [47]. The amount of work required to separate the friction-exposed materials is measured by apparent frictional energy density (AFED) [42]. The AFED ($e_{R}^{*}$) is the frictional work performed ($W_{R}$) per wear volume ($V_{V}$); Equation (1) [48,49].

(1) $$e_{R}^{*}=\frac{W_{R}}{V_{V}}$$

Frictional force ($F_{f}$) and the distance traveled by the rotating ball (*D*) were used to calculate the frictional work performed ($W_{R}$); Equation (2).

(2) $$W_{R}=F_{f}\times D$$

The distance traveled by the rotating ball was given by Equation (3):

*D* = 2*πa* × *rpm* (3)

where

a is the radius of the ball (6.35 mm) and rpm is the speed of the rotating ball (1200 rpm). The frictional force ($F_{f}$) was found from the normal force ($F_{n}$) and the coefficient of friction (μ); Equation (4) [50].

(4) $$F_{f}=F_{n}\times \mu$$

The volume of the wear scar is related to the wear scar diameter (d) by Equation (5) [51]:

(5) $$V_{v}=c_{1}d^{4}-c_{2}Wd$$

where

W is the load in kg, $V_{v}$ is in mm^3^, and *d* is in mm. The values of the constants for the steel ball of radius 6.35 mm are *c*_1_ = $1.55\times 10^{-2}$ mm^−1^ and *c*_2_ = $1.07\times 10^{-5}$ mm^2^/kg [46]. The linear wear intensity ($I_{h}$) according to Fleischer’s fundamental equations [52] can be found in Equation (6):

(6) $$I_{h}=\frac{\tau_{R}}{e_{R}^{*}}$$

where

$\tau_{R}$ is the frictional shear stress, which was found by Equation (7):

(7) $$\tau_{R}=\mu \cdot P_{a}$$

where

$P_{a}$ is the contact pressure and was found from Equation (8) [53]:

(8) $$P_{a}=\frac{L}{\pi d^{2}/4}$$

where

*L* is the contact load. The Contact load *L* can be found from the applied load by Equation (9) [53,54]:
  (9) $$L=\frac{1}{\sqrt{6}}W$$

According to Fleischer, the basic graph of the energy equation contains five regimes (0–4) and each regime has its own characteristic friction and wear states; Table 5. The friction states were identified depending on the lubrication regime the lubricant falls under.

It should be noted that the application of Fleischer’s energy-based wear model to four-ball tribological configurations has not been universally validated, and in the present study it is employed as a comparative and interpretative framework rather than a predictive model for component-level performance.

### 2.6. Energy Consumption Test Using Modified Brugger Machine

**Figure 6:**
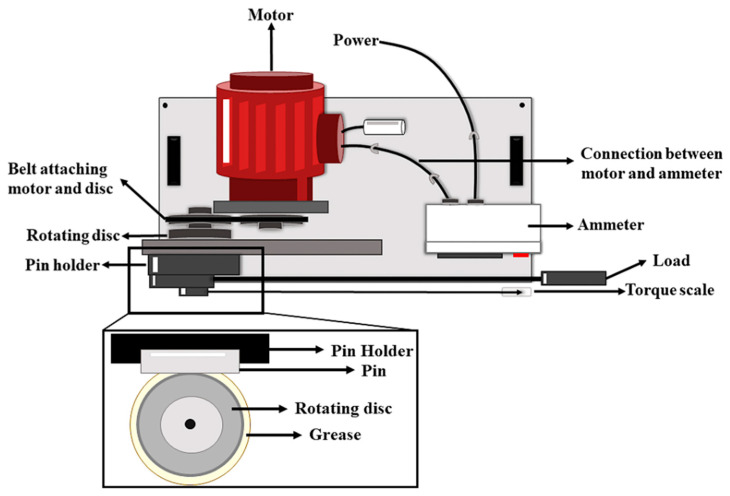
Current consumption tester.

A schematic illustration of a modified Brugger machine for energy consumption analysis is given in Figure 6. The energy consumption in the presence of the greases was measured using a modified Brugger test rig that was constructed internally [39]. A revolving disk was coated with the test grease. After being inserted into the pin holder, an EN-31 pin was pressed 10 Nm^−1^ against the disk. Figure 6 shows that the pin and the revolving disk made contact with two crossed cylinders of varying sizes. At 860 rpm, the disk rotated. The test took one minute to complete. The revolving disk was attached to a motor. A machine-mounted analytical ammeter was used to measure the current used by the motor while the disk rotated. The steady value was recorded.

### 2.7. Characterizations

The balls used for the tribological tests were analyzed using a scanning electron microscope (SEM) (Make: Zeiss, Zena, Germany, Model: EVO 18) to study the morphology of the worn surfaces. A Raman spectroscope (Make: WiTec Model, Ulm, Germany: Alpha 300) was used to identify the tribofilm formation on the mating surfaces.

## 3. Results and Discussion:

### 3.1. Thermal Studies of the Grease Samples

**Figure 7:**
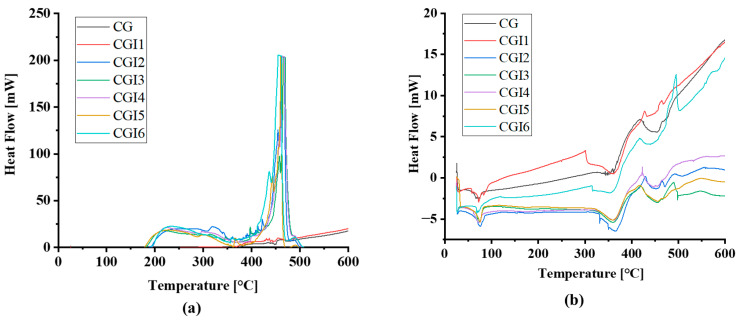
DSC of the grease samples in (**a**) nitrogen and (**b**) oxygen environments.

The thermal stability of the prepared grease samples was analyzed using thermo-gravimetric analysis (TGA) and differential scanning calorimetry (DSC) in both nitrogen (N_2_) and oxygen (O_2_) environments. Figure 7a shows the DSC curves of the greases under a nitrogen atmosphere, while Figure 7b presents the results under an oxygen atmosphere. Under N_2_, the base grease (CG) and the sample with the lowest illite content exhibit relatively stable thermal behavior, with no significant exothermic degradation up to 600 °C. In contrast, greases containing higher concentrations of illite (CGI2–CGI6) have distinct exothermic peaks around 420–480 °C (Figure 7a). These peaks suggest that illite promotes non-oxidative thermal decomposition due to the de-hydroxylation of illite in that temperature range [55]. Under an O_2_ atmosphere (Figure 7b), all samples exhibit earlier and more pronounced exothermic responses, with degradation initiating at lower temperature ranges. These observations highlight the role of illite in grease systems, enhancing catalytic degradation under inert conditions and further amplifying oxidative instability in oxidative environments. This also shows the usage limit of illite greases was 400 °C. Previous studies [56,57] on clay-based lubricant additives suggest that oxidative instability can be reduced through surface modification of clay particles, incorporation of antioxidant packages, or hybridization with oxidation-inhibiting additives. Such approaches may suppress catalytic activity at the clay–oil interface and delay oxidative degradation.

**Figure 8:**
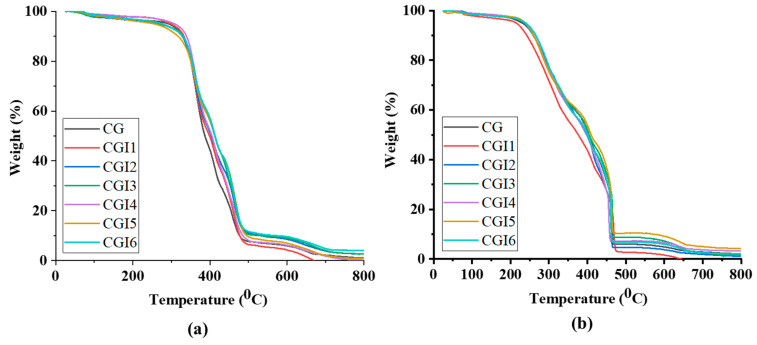
TGA of the grease samples in (**a**) nitrogen and (**b**) oxygen environments.

Thermal stability was assessed by thermo-gravimetric analysis and two fixed-residue markers were isolated: temperatures at which 50% and 75% mass loss happen (Figure 8). With an inert atmosphere (N_2_), decomposition occurs mainly through pyrolysis and char formation; therefore, the 50%-remaining and 25%-remaining markers appear at comparatively high temperatures (Table 6). The degradation of the basic castor oil is the cause of the sharp decline in mass or steepness at around 3000 °C that was seen in the overall form of the graph. This is supported by the fact that the hydroxyl triacylglycerols (TAGs) in castor oil have been reported to exhibit a notable decrease at 300 °C [58]. Under an oxidative environment (O_2_), the initiation and development of mass loss occurs at lower temperatures due to faster oxidation.

### 3.2. Wear and Frictional Analysis of the Grease Samples

**Figure 9:**
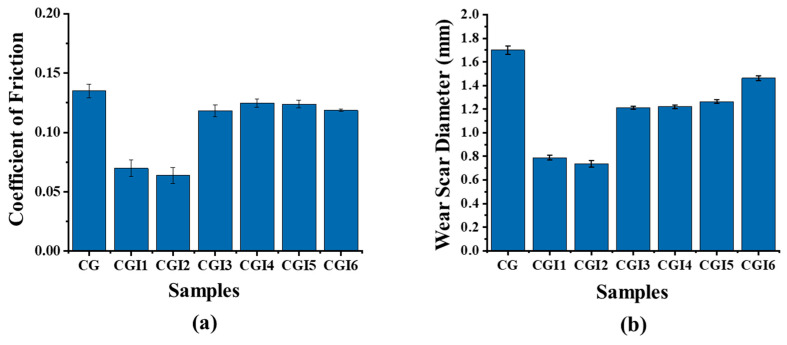
(**a**) COF and (**b**) wear scar diameter of the grease samples.

The tribological performance of calcium grease (CG) and its composites with varying concentrations of illite was evaluated in terms of coefficient of friction (COF) and wear scar diameter (WSD) using a four-ball tribometer following the ASTM D5183 and ASTM D2266 standards, respectively (Figure 9).

**Figure 10:**
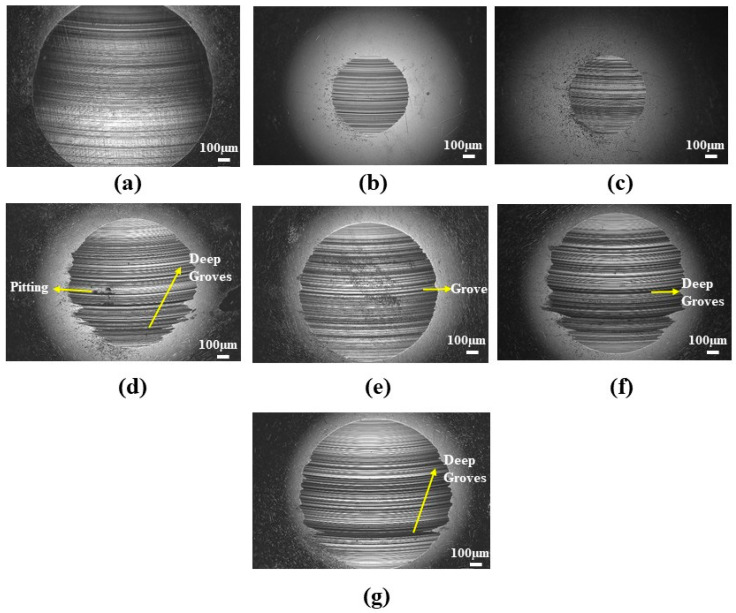
Wear scars of the balls lubricated with greases (**a**) CG, (**b**) CGI1, (**c**) CGI2, (**d**) CGI3, (**e**) CGI4, (**f**) CGI5, and (**g**) CGI6.

The base grease (CG) exhibited the highest COF (0.135 ± 0.004) and the largest wear scar diameter (1.7 ± 0.04 mm), indicating limited friction-reducing and anti-wear capabilities. With the addition of illite, both the COF and WSD values decreased significantly. CGI2 recorded the lowest COF (0.06379 ± 0.006), which was 53% lower than that of the base grease. However, with a further increase in the additive percentage, the value of the COF kept increasing. CGI2 showed the smallest WSD (0.736 ± 0.03 mm), which was 57% smaller than that of the base grease, suggesting it offered the best anti-wear protection. These improvements can be attributed to the layered structure of illite [59] (Hamza et al., 2023), which provides a solid lubricating effect and helps form a protective film on the contact surface. The microscopic images of the wear scars are shown in Figure 10.

**Figure 11:**
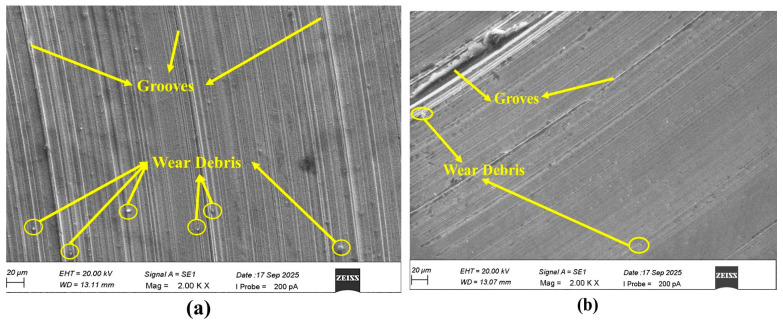
Scanning electron microscopy images of the wear scar of the balls lubricated with greases (**a**) CG and (**b**) CGI2.

The presence of wear tracks in the ball surface shows that the abrasive wear mechanism occurred during the four-ball anti-wear test (Figure 11).

These wear tracks were found to be smaller for the grease with low concentrations of illite, and deep grooves and small micro-pitting were found on the ball surfaces with a higher concentration of illite. Additionally, wear debris was found adhered to the ball surfaces. The build-up and periodical release of this wear debris would initially increase the COF. During the surface interaction process in sliding wear, the materials either move to the wear track edges (plastic deformation) or are pushed to the wear tracks by the counter body, later resulting in wear debris [60]. As these build-up materials (wear debris) are released, the resistance decreases and hence reduces the COF. The adherence of the wear debris on the ball surfaces would also increase their roughness, leading to the formation of plastic deformations [60] and affecting the lubrication regime as seen in Section 3.3. The presence of grooves as well as adhered debris indicated a mixed wear phenomenon (abrasive–adhesive), similar to that reported by Escherova et al. [60]. Table 7 shows the changes in the surface roughness parameters (Ra and Rq) before and after the wear test. The base grease exhibits a significantly higher increase in roughness (Ra = 2.96 ± 0.41 µm and Rq = 3.56 ± 0.73 µm) compared to the greases containing illite as an additive. Among the illite-based greases, CGI2 exhibited the lowest increase in roughness (Ra = 0.73 ± 0.24 µm and Rq = 0.98 ± 0.39 µm), indicating effective tribofilm formation and enhanced surface protection against wear damage. With an increase in illite concentration, the surface roughness increased, which resulted in high surface damage.

### 3.3. Apparent Frictional Energy Density (AFED) Wear Analysis for the Grease Samples

**Figure 12:**
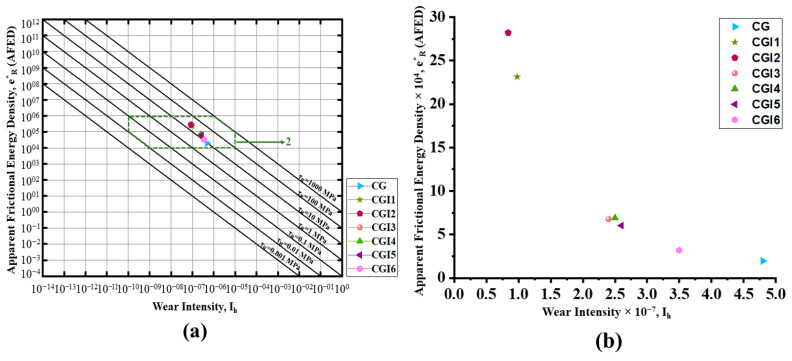
(**a**) Basic energy equation of wear graph for the grease samples; (**b**) apparent frictional energy density vs. wear intensity graph.

In order to evaluate the lubrication performance and wear behavior of each grease sample, Fleischer’s energy-based wear model was used to calculate the apparent frictional energy density (AFED). Table 8 displays the computed AFED, wear intensity, and frictional shear stress values. To assess the lubrication regime for every sample, a plot was created between AFED and wear intensity. According to the graph (Figure 12a), all samples are in regime 2, or mixed friction, where the tribological interaction is dominated by both plastic deformation and rheological effects. Among the tested grease samples, CG had the lowest AFED value (19,672.29 J/mm^3^), but it also had the highest linear wear intensity (4.83969 × 10^−7^), suggesting that it was not very effective at transforming frictional energy into protective mechanisms at the contact interface. On the other hand, the wear and frictional properties were greatly enhanced by the addition of illite. CGI2 outperformed CG in terms of AFED (282,272 J/mm^3^) and wear intensity (8.49 × 10^−8^). CGI4 demonstrated a lower AFED (31,872.25 J/mm^3^) and a higher wear volume (0.0698 mm^3^), despite having a low COF (0.1186), indicating an instability or a lower load-carrying capacity in the protective film. In comparison to the base grease, the energy efficiency and wear resistance of the grease formulations were greatly improved by the addition of illite, particularly CGI2 (Figure 12b). Compared to CG, CGI2 decreased the wear intensity by 82%. These findings demonstrate that the illite-containing greases have better tribological performance in a mixed lubrication regime because they make better use of frictional energy to reduce wear.

### 3.4. Statistical Data Analysis for the Anti-Wear Properties and Coefficient of Friction of the Grease Samples

As shown in Table 8, the data were statistically examined using one-way ANOVA to guarantee the validity of the experimental findings. ANOVA helps to examine the effects of additives in the experimental runs [39,61]. The *p*-value is fixed at 0.05 [62]. The results of the one-way ANOVA analysis confirmed a statistically significant difference among the tested groups. For the coefficient of friction (COF) values of additivated greases compared with the base grease, the *p*-value was 1.79 × 10^−10^, indicating a highly significant effect of additive incorporation (Table 9). Similarly, for the wear scar diameter (WSD), the comparison with the base grease yielded a *p*-value of 4.84 × 10^−9^, confirming the effectiveness of additives in reducing wear (Table 9).

Further, when additivated greases with different concentrations were compared among themselves, highly significant differences were observed for both COF (*p* = 4.86 × 10^−32^) and WSD (*p* = 5.20 × 10^−26^) (Table 10). In the tribological tests, CGI2 exhibited the most favorable performance and the statistical data analysis proved its significantly superior tribological behavior compared to both the base grease and the other additive concentrations.

### 3.5. Analyzing Extreme-Pressure Properties of the Grease Samples

The extreme-pressure properties of the grease samples were determined using the ASTM D2596 method. The weld load is the force at which the ball’s local temperature increases beyond its melting temperature, causing the balls to become welded together [63]. The Hertz line, Hertz diameter, last non-seizure load (LNSL), initial seizure load (ISL), and weld load (WL) were found to evaluate the load-carrying capacity of the samples. The Hertz diameter can be found by using the formula seen in Equation (10) [64]:

(10) $$\;\;\;\;D_{h}=0.0873P^{\frac{1}{3}}$$

where

*P* is the load given.

Load–wear index (*LWI*) is a measure of a lubricant’s ability to withstand high loads without causing excessive wear on the surface. It was found using Equation (11) [65],

(11) $$\;\;\;\;LWI=\frac{\sum CL}{10}$$

where

∑*CL* is the sum of the Corrected Loads (*CL*) for ten applied loads preceding the weld load, which can be calculated by Equation (12) [65],

(12) $$\;\;\;\;CL=\frac{PD_{h}}{d}$$

Apart from the Hertzian contact diameter, several simplified analytical models based on contact geometry and assumptions of plastic deformation have been reported to estimate wear scar diameter in a four-ball tribological configuration. Menga et al. [66] developed models from geometric considerations of spherical contact and provided a first-order estimation of wear scar diameter under given load conditions. Although such analytical approaches offer useful baseline predictions, they do not account for the complex physicochemical effects of lubricants, additives, tribofilm formation, and third-body interactions. Thus, the wear scars from the experiments not only reflect the contact mechanics, but also consider the illite-induced lubrication mechanisms, which purely analytical models cannot fully capture.

**Figure 13:**
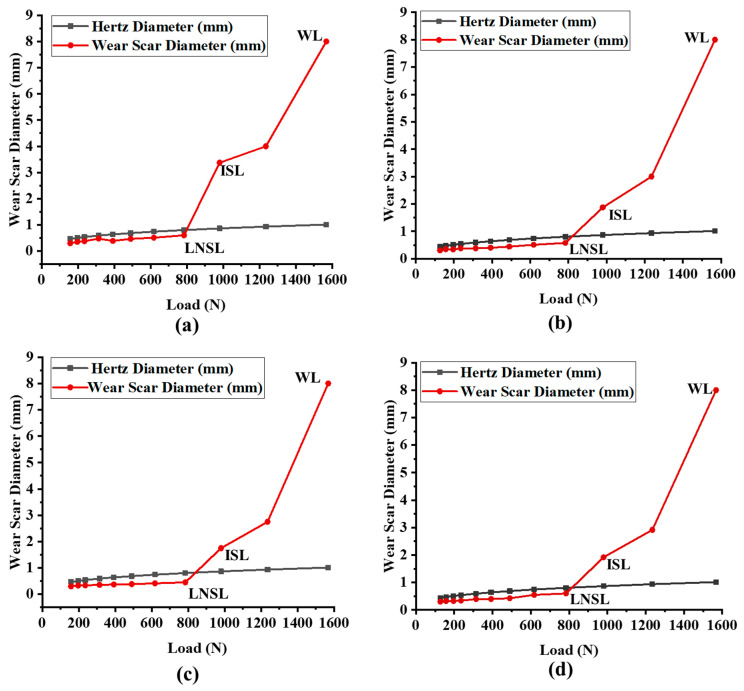

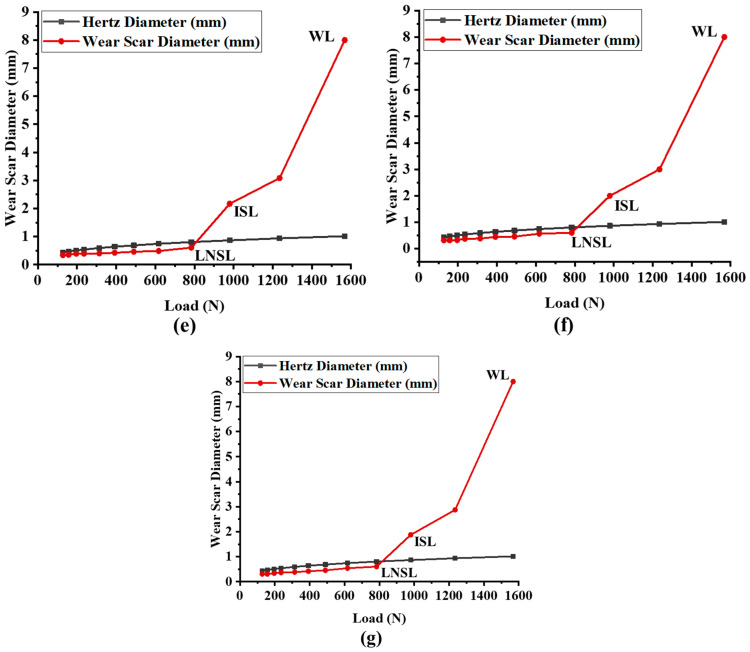
Wear scar diameter vs. load curve for (**a**) CG, (**b**) CGI1, (**c**) CGI2, (**d**) CGI3, (**e**) CGI4, (**f**) CGI5, and (**g**) CDI6.

**Figure 14:**
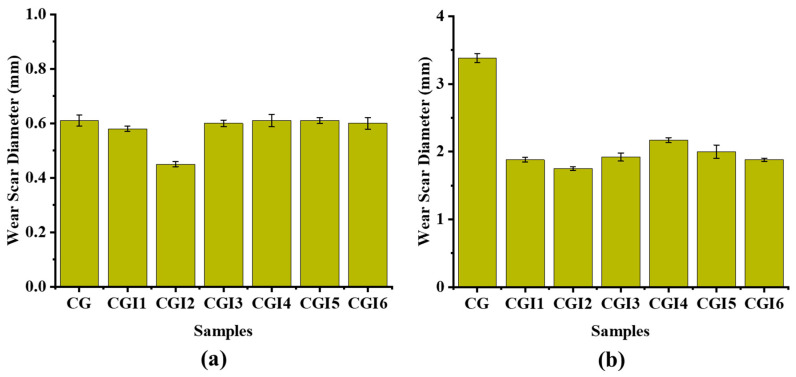
Wear scar diameter at (**a**) LNSL and (**b**) ISL of the grease samples.

**Figure 15:**
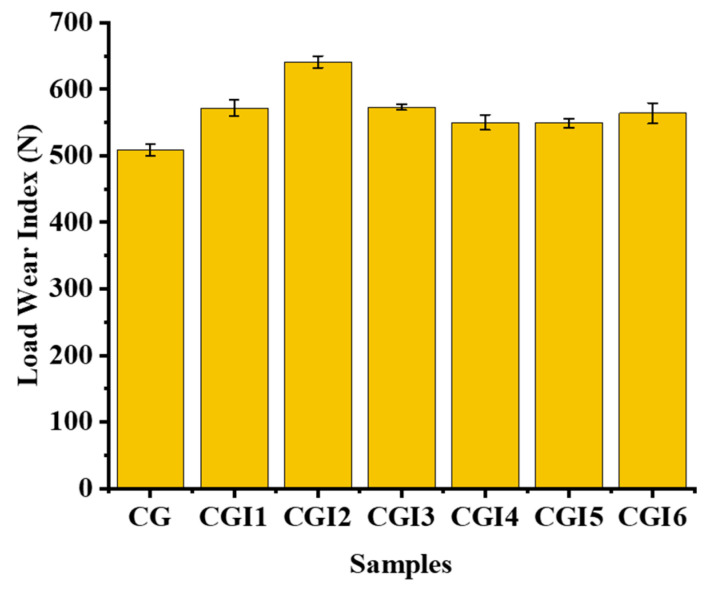
Load–wear index of the grease samples.

**Figure 16:**
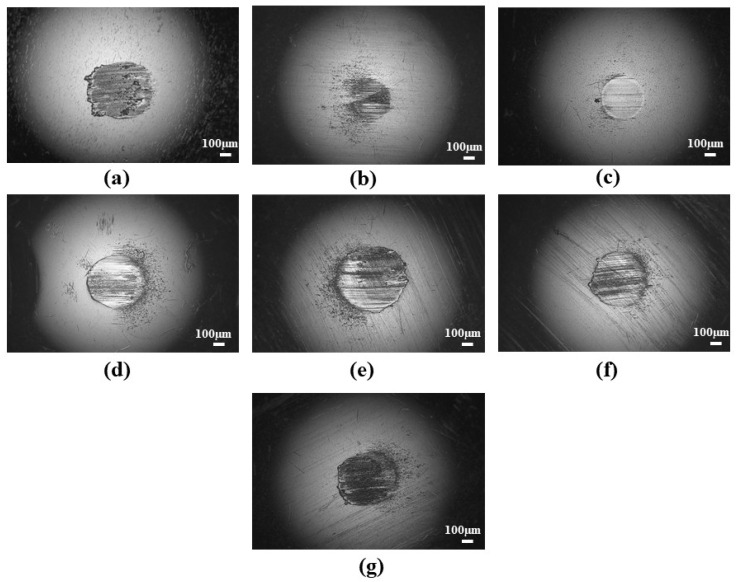
Optical microscopy images of the surfaces of balls at LNSL with grease (**a**) CG, (**b**) CGI1, (**c**) CGI2, (**d**) CGI3, (**e**) CGI4, (**f**) CGI5, and (**g**) CGI6.

**Figure 17:**
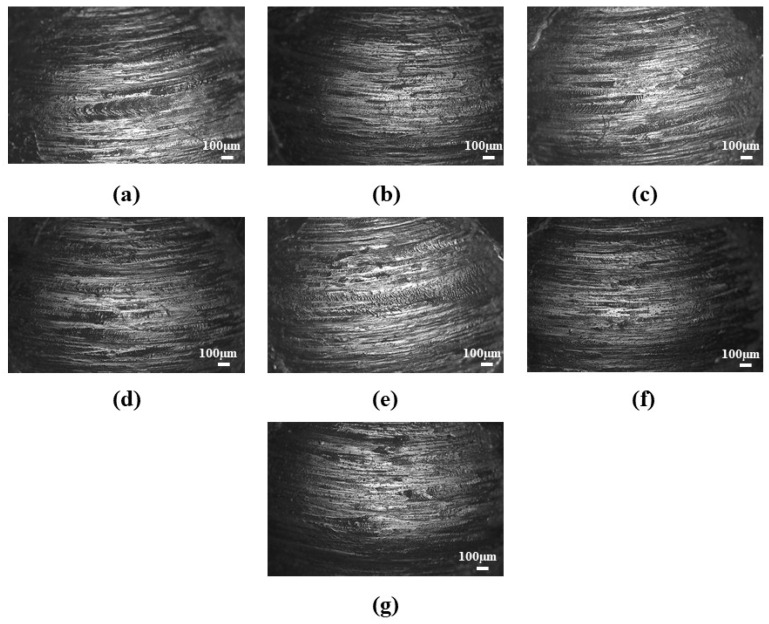
Optical microscopy images of the surfaces of balls at ISL with greases (**a**) CG, (**b**) CGI1, (**c**) CGI2, (**d**) CGI3, (**e**) CGI4, (**f**) CGI5, and (**g**) CGI6.

The weld load (WL) values were observed to be the same for all of the tested grease samples (1568 N) (Figure 13). The last non-seizure load (LNSL) of all the tested grease samples was 784 N (Figure 14a). While the load-carrying capacity at this stage was the same, the wear scar diameter (WSD) differed. Among them, CGI2 exhibited the lowest wear scar diameter of 0.4524 ± 0.009 mm, which is 23% smaller than CG (0.61 ± 0.02 mm). This reduction in scar size for CGI2 indicates that it provided a more effective protective film on the contact surfaces. The initial seizure load (ISL) of all of the tested grease samples was 980 N (Figure 14b). However, CGI2 produced the smallest wear scar with a diameter of 1.75 ± 0.026 mm, which is 48% smaller than that observed for CG (3.375 ± 0.065 mm). This suggests that even when the seizure onset for the tested grease samples was the same, CGI2 minimized the wear. The load–wear index (LWI) of CGI2 was 641 ± 8.62 N, which is 21% higher than 509 ± 9.1 N for CG (Figure 15). It was observed that the load-carrying capacity reduced with further increases in additive concentration (Figure 15). This trend can be explained by the fact that a large quantity of clay additive results in agglomeration, which prevents the rolling effect of the additive particles. These agglomerated particles will further cause abrasive wear on the mating surfaces [67]. Figure 16 and Figure 17 show the optical microscopic images of LNSL and ISL, respectively.

As observed from the surface images under high-load and extreme-pressure conditions, the wear scars exhibit significant unidirectional grooves (Figure 10, Figure 16 and Figure 17). These kinds of morphologies are predominantly characteristic of abrasive wear, highly influenced by severe metal–metal contact. Thus, these resulting wear scars are highly anisotropic, and the directional roughness of the contact surfaces influences the resulting coefficient of friction measurement. Consequently, the reported COF under these conditions should be interpreted as a qualitative indicator of tribological performance rather than that of absolute isotropic roughness conditions [68].

### 3.6. Energy Consumption Test

**Figure 18:**
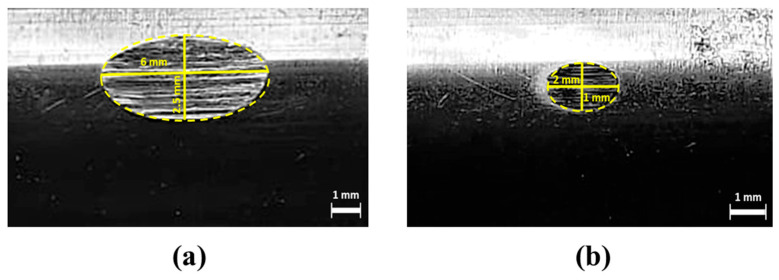
Photographs of the wear scar of the pins for (**a**) CG and (**b**) CGI2.

An energy consumption test was used to evaluate the base grease and the optimized greases’ energy efficiency. This test indicates how much current the motor attached to the rotating disk uses. The grease between them determined how easily the rotating disk rotated because it was in contact with the pin at a force of 10 N for 1 min. The average of three readings is shown in Table 11 and Figure 18 shows a photograph of the wear scars. The current consumption and the wear scar diameter of CG were 1.7 ± 0.05 A and 4.25 ± 0.05 mm, respectively (Figure 18). The addition of 0.1% of illite reduced the current consumption by 6% and the wear scar diameter by 65% (Table 11). This reduction in current consumption is due to the reduced friction observed in the ASTM D5183 test, and the significant reduction in wear dimension indicates the anti-wear property of the illite.

### 3.7. Lubrication Mechanism

**Figure 19:**
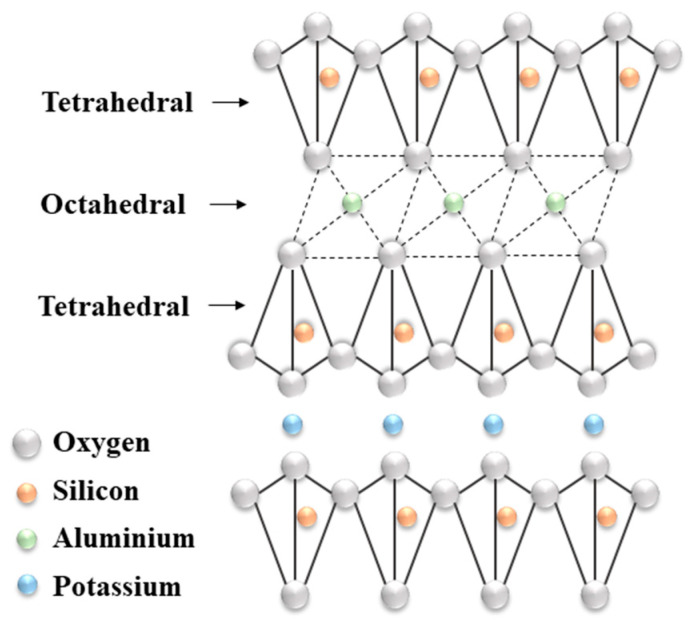
Structure of illite.

The structure of illite is layered (Figure 19) [69]. Atoms in the same plane are bound by strong covalent bonds, while the layers of the illite are bound by weak forces such as the electrostatic force and the Van der Waals force, as in other clay minerals [70]. These weak forces between the layers facilitates easy sliding when the material is used as an additive in lubrication [71]. The interlayer spacing of illite is about 1.0 nm [72].

During sliding contact, fine illite particles can become entrapped in the contact zone and form a thin, protective layer (tribofilm) on the surface. This film acts as a physical barrier, minimizing direct metal-to-metal contact and thereby reducing adhesive wear [73]. Illite particles, particularly when finely milled, can behave like microscopic rolling elements between asperities, facilitating smoother movement. This rolling action further reduces the friction that leads to wear [67]. The illite particles contribute to asperity filling, leading to improved load distribution across the contact surface. This reduces localized pressure points and abrasive interactions, lowering both wear and COF over time [74]. Illite is thermally stable and chemically inert under operating temperatures typical in grease applications [75]. Its presence enhances the grease’s resistance to thermal degradation and helps maintain consistent lubrication, indirectly reducing wear caused by heat-induced breakdown of the lubricant.

**Figure 20:**
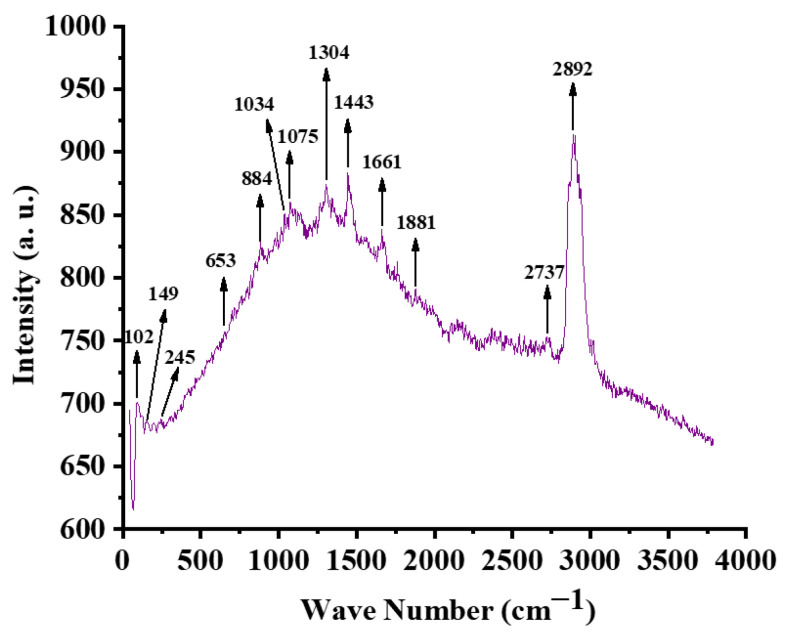
Raman spectrum of wear scar lubricated with illite grease.

**Figure 21:**
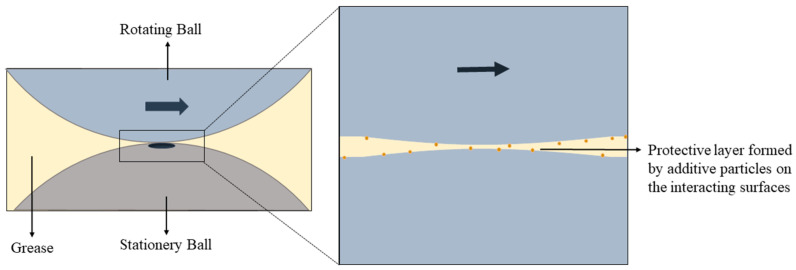
A protective layer formed by the illite particles on the interactive surfaces.

The Raman spectrograph of the worn surface of the ball lubricated with CGI2 shows the characteristic spectrographic range for illite (Figure 20). The peak at 149 cm^−1^ represents the lattice vibrations. The peak at 245 cm^−1^ represents the bending vibrations of Si-O bonds. The peak at 1034 cm^−1^ represents the stretching vibrations of Si-O bonds. It also shows the characteristic peaks of the oil. The peaks at 1075 cm^−1^ and 1304 cm^−1^ represent the ester linkages in the castor oil’s triglycerides. The peak at 1443 cm^−1^ represents the fatty acid components. The peak at 1661 cm^−1^ represents the carboxyl groups in the fatty acids. The peaks at 2737 cm^−1^ and 2892 cm^−1^ represent the fatty acid components. The peaks at 653 cm^−1^ and 884 cm^−1^ represent the iron oxides [76]. This iron oxide layer works as a sacrificial protective film that takes the wear instead of the steel surface. It prevents direct metal-to-metal contact, allows easier sliding, and helps in reducing friction [77]. These spectrograph characteristics give strong evidence for the creation of a protective tribolayer on the ball surface, which resulted in reduced wear (Figure 21). This supports the observation that illite is a potential biocompatible anti-wear additive.

**Figure 22:**
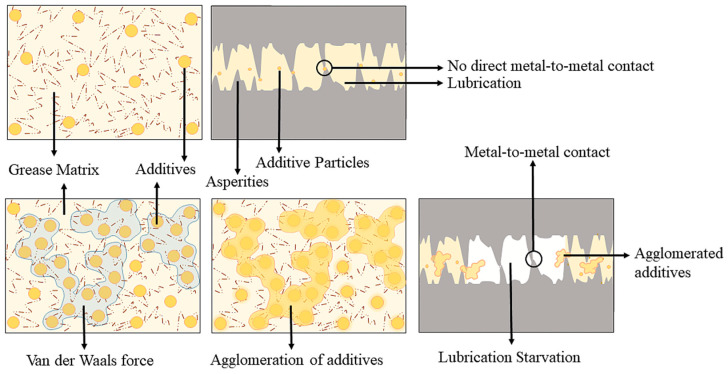
Agglomeration of illite particles.

In the extreme-pressure test, although the weld load remained unchanged for both greases, the improved wear resistance and higher LWI indicate that illite plays a crucial role in sustaining loads without severe surface damage. This shows that the mechanism of illite differs from that of conventional sulfur- or phosphorus-based EP additives, which enhance seizure load and weld resistance through chemical reactions [78]. In contrast, illite enhances tribological performance primarily through physical surface protection, layered sliding, and load distribution effects, as well as by forming a weakly bonded tribofilm under repeated sliding, as confirmed by Raman spectroscopy analysis. However, a trend of increasing COF and wear was observed during the tests. This is due to the agglomeration of illite particles, which blocks lubrication between the contacting surfaces [3]. This agglomeration causes increased wear and friction [79,80] (Figure 22). This shows that at lower concentrations, illite acts as a good anti-wear additive.

## 4. Conclusions

In this study, calcium grease was made from castor oil as a base oil and illite was used as an additive at different concentrations. The produced greases were studied for their thermal properties, anti-wear properties, frictional properties, and energy consumption. The results were compared to find the optimal additive concentration. The following conclusions are formed.

1. Thermo-gravimetric analysis showed that under an inert N_2_ atmosphere, decomposition of the grease formulations proceeded mainly through pyrolysis with higher residual mass, resulting in elevated 50%- and 25%-mass-remaining temperatures, particularly for the CGI5 and CGI6 samples. In contrast, under oxidative O_2_ conditions, mass loss was initiated earlier and progressed more rapidly due to oxidation, leading to lower 50%- and 25%-mass-remaining temperatures. These results indicate that illite contributes to improved late-stage thermal stability under inert conditions by enhancing inorganic residue, while simultaneously promoting greater oxidative reactivity under O_2_.
2. Under N_2_, the base grease (CG) and the grease with the lowest illite content displayed stable thermal behavior up to 600 °C, whereas samples with higher illite concentrations (CGI2–CGI6) showed distinct exothermic peaks at 420–480 °C, indicating that illite promotes non-oxidative thermal decomposition due to the de-hydroxylation of illite. Under O_2_, all samples exhibited earlier and stronger exothermic responses, with degradation initiating at lower temperatures, demonstrating that illite not only enhances catalytic degradation under inert conditions but also amplifies oxidative instability. These findings suggest that the practical thermal usage limit of illite-containing greases is restricted to approximately 400 °C.
3. The tribological performance of calcium grease (CG) is significantly improved with the addition of illite. The optimal concentration of illite is found to be 0.1 wt.%, CGI2. It provided the most effective friction reduction (COF 53% lower than the base grease) and anti-wear protection (57% lesser wear than the base grease). Beyond this optimal concentration, the anti-friction and anti-wear properties begin to decrease as a result of lubricant starvation due to the agglomeration of additive particles.
4. The statistical significance of the experimental test results was confirmed by performing an ANOVA test which gave a *p*-value less than 0.05, rejecting the null hypothesis.
5. Raman spectroscopy of the worn surface lubricated with CGI2 confirms the presence of both illite and oil components. The detection of characteristic peaks for illite proves the formation of a protective tribolayer. This shows that the observed reduction in wear is because of the illite forming a protective film on the surface.
6. The study, using Fleischer’s energy-based wear model, indicates that all of the grease samples operated within the mixed friction regime. While the base grease (CG) has the lowest apparent frictional energy density (AFED) (19,672 J/mm^3^), it also exhibited the highest wear intensity (4.8 × 10^−7^), signifying poor protective capabilities. The addition of illite significantly improves the tribological properties. CGI2 has the highest AFED (282,273 J/mm^3^) and lowest wear intensity (8.4 × 10^−8^), suggesting superior energy dissipation and wear resistance compared to the other tested samples. The conclusions drawn from Fleischer’s energy-based wear analysis should therefore be interpreted in a comparative sense, as the model is not yet fully validated for four-ball tribological configurations.
7. In the extreme-pressure test, while the tested samples showed equal load-carrying capacities at the last non-seizure load (LNSL) (784 N), initial seizure load (ISL) (980 N), and weld load (WL) (1568 N), their anti-wear performance was significantly different. The CGI2 composite consistently showed smaller wear scar diameters (WSD) in each load stage, demonstrating a more efficient protective film. Also, CGI2 (641 ± 8.62 N) had a 21% greater load–wear index (LWI) than CG (509 ± 9.1 N), which shows that the illite additive has better extreme-pressure (EP) performance and wear resistance. Load-carrying ability was observed to reduce with further increases in additive concentration.
8. The energy consumption test demonstrated that CGI2 (1.6 ± 0.05 A) significantly improved the energy efficiency compared to CG (1.7 ± 0.05 A). The addition of just 0.1 wt.% illite reduced the motor’s current consumption by 6%, indicating a lower coefficient of friction. Also, CGI2 (1.5 ± 0.2 mm) had a 65% smaller wear scar diameter than CG (4.25 ± 0.05 mm).
9. The base calcium grease exhibited a worked cone penetration of 257 dmm (, corresponding to an NLGI grade 2 consistency. The measured dropping point of the base grease was 80 ± 2 °C, while the illite-containing greases showed slightly reduced dropping points in the range of 70–72 °C, attributed to interactions between illite particles and the calcium soap matrix.

Though there was a decrease in the drop point of the calcium hydroxide grease, the present work exhibits illite as an eco-friendly, cost-effective anti-wear additive that can reduce friction, wear, and energy consumption when used in an optimal concentration. The future scope of this study involves an evaluation of the rheological and further tribological performance of the present grease alongside commercial grease and calcium sulfonate grease. Additionally, other tests such as a corrosion test and a water washout test will be planned in the future. Furthermore, while the four-ball tribological tests provide reliable comparative evaluation and mechanistic understanding, future work will focus on validating the optimized grease formulation under bearing-level tests, including rolling contact fatigue, temperature rise, and long-term durability, to further assess its industrial applicability.

Although standardized four-ball tribological tests -) provide reliable comparative insights into friction, wear, and extreme-pressure behavior, they do not constitute a direct prediction of grease performance in rolling-element bearings. Bearing applications involve complex rolling–sliding contacts, grease replenishment dynamics, and fatigue-driven failure modes that are not captured by four-ball configurations. Therefore, future work will focus on bearing-level validation, including rolling contact fatigue, temperature rise, grease life, and long-term durability tests, to further assess the industrial applicability of the optimized grease formulation.

## Figures and Tables

**Table 1:** Viscosity, total acidic number (TAN), and total base number (TBN) of the castor oil.

Parameters	Unit	Value
Viscosity at 40 °C	cSt	194
Viscosity at 80 °C	cSt	32
Flash point	°C	270
Fire point	°C	300
TAN	mgKOH/g	1.96
TBN	MgKOH/g	0.16

**Table 2:** Elemental composition of the balls.

Elements	Carbon	Manganese	Silicon	Sulphur	Phosphorous	Chromium
Weight percentage (%)	0.925	0.321	0.271	0.014	0.010	1.441

**Table 3:** Drop points of the grease samples.

Samples	CG	CGI1	CGI2	CGI3	CGI4	CGI5	CGI6
Drop Point, °C	80 ± 2	70 ± 2	70 ± 2	72 ± 3	72 ± 2	71 ± 2	70 ± 3

**Table 4:** Parameters to evaluate the tribological properties of the grease samples.

Test	ASTM Standard	Temperature (°C)	Load (N)	Speed (rpm)	Duration
Anti-wear test	ASTM D2266 [44]	75 ± 2	392	1200 ± 60	60 ± 1 min
Coefficient of friction	ASTM D5183 [45]	75 ± 2	392	600 ± 60	60 ± 1 min
Extreme-pressure test	ASTM D2596 [46]	Room temperature	As per standard	1760 ± 60	10 s

**Table 5:** Friction and wear states for corresponding regimes.

Regime	Friction State	Wear State	Process Parameter		
			$e_{R}^{*}$ (J/mm^3^)	$\tau_{R}$ (MPa)	$I_{h}$
0	Fluid friction	Zero wear	10^10^–10^7^	10^0^–10^−3^	<10^−13^
1	Fluid/Mixed friction	Level 1	10^9^–10^6^	10^3^–10^−3^	10^−13^–10^−7^
2	Mixed friction	Level 2	10^6^–10^4^	10^3^–10^−3^	10^−11^–10^−5^
3	Solid friction	Level 3	10^6^–10^2^	10^3^–10^−2^	10^−10^–10^−3^
4	Solid friction	Level 4	10^4^–10^1^	10^3^–10^−2^	10^−8^–10^−3^

**Table 6:** Degradation temperature of the grease samples in nitrogen and oxygen environments.

Sample	Degradation Temperature (°C)			
	N_2_		O_2_	
	50%	25%	50%	25%
CG	387.15	442.867	404.517	461.4
CGI1	399.133	452.1	376.2	455.267
CGI2	403.55	460.833	398.25	455.667
CGI3	413.333	462.467	404.65	463.5
CGI4	405.183	454.15	399.033	453.75
CGI5	412.6	465.35	409.7	461.75
CGI6	413.05	464.233	400.933	458.717

**Table 7:** Surface roughness parameters (Ra and Rq) of the ball before and after the anti-wear test for different grease samples.

Samples	Ra (µm)		Rq (µm)	
	Before Test	After Test	Before Test	After Test
CG	0.10 ± 0.001	2.96 ± 0.41	0.12 ± 0.002	3.56 ± 0.73
CGI1	0.09 ± 0.0005	0.99 ± 0.39	0.14 ± 0.004	1.23 ± 0.45
CGI2	0.11 ± 0.0015	0.73 ± 0.24	0.14 ± 0.001	0.98 ± 0.39
CGI3	0.11 ± 0.005	2.49 ± 0.46	0.12 ± 0.005	2.77 ± 0.39
CGI4	0.11 ± 0.006	2.47 ± 0.33	0.14 ± 0.002	2.95 ± 0.28
CGI5	0.122 ± 0.001	2.62 ± 0.27	0.12 ± 0.002	2.98 ± 0.31
CGI6	0.11 ± 0.003	2.91 ± 0.37	0.11 ± 0.001	3.31 ± 0.52

**Table 8:** Tribological process parameters of the grease samples.

Samples	WSD (mm)	COF	AFED (J/mm^3^)	Linear Wear Intensity	Friction Shear Stress (N/mm^2^)
CG	1.7	0.135	19,672.28692	4.83969 × 10^−7^	9.52
CGI1	0.789	0.0699	231,295.2929	9.89447 × 10^−8^	22.88
CGI2	0.736	0.0637	282,272.4501	8.49086 × 10^−8^	23.96
CGI3	1.21	0.1183	67,847.64891	2.42726 × 10^−7^	16.47
CGI4	1.22	0.1247	69,175.29063	2.46849 × 10^−7^	17.08
CGI5	1.26	0.1239	60,324.64987	2.63677 × 10^−7^	15.91
CGI6	1.46	0.1186	31,872.25494	3.55797 × 10^−7^	11.34

**Table 9:** ANOVA *p*-values for the COF and anti-wear test results of all grease samples.

Sample	*p*-Value
Coefficient of Friction	1.79 × 10^−10^
Wear Scar Diameter	4.84 × 10^−9^

**Table 10:** ANOVA *p*-values for the COF and anti-wear test results of CGI samples.

Sample	*p*-Value
Coefficient of Friction	4.86 × 10^−32^
Wear Scar Diameter	5.20 × 10^−26^

**Table 11:** Current consumption and wear scar dimension of the samples.

Samples	CG	CGI2
Average current consumed (A)	1.7 ± 0.05	1.6 ± 0.05
Average scar diameter (mm)	4.25 ± 0.05	1.5 ± 0.2

## Data Availability

The original contributions presented in this study are included in the article. Further inquiries can be directed to the corresponding authors.
